# Age and gender correlation of gonial angle, ramus height and bigonial width in dentate subjects in a dental school in Far North Queensland

**DOI:** 10.4317/jced.52683

**Published:** 2016-02-01

**Authors:** Jodi Leversha, Glen McKeough, Adriana Myrteza, Hannah Skjellrup-Wakefiled, Jordan Welsh, Amar Sholapurkar

**Affiliations:** 1 BDS, College of Medicine and Dentistry, James Cook University, 14-88 McGregor Rd, Smithfield. Cairns. QLD. 4878. Australia; 2 BDS, MDS, College of Medicine and Dentistry, James Cook University, 14-88 McGregor Rd, Smithfield. Cairns. QLD. 4878. Australia

## Abstract

**Background:**

This study aimed to determine if mandibular parameters (gonial angle, bigonial width and ramus height) measured from panoramic radiographs, can be used to determine a correlation with an individual’s age and gender in dentate subjects in Far North Queensland.

**Material and Methods:**

The study utilised 2699 randomly selected panoramic radiographs of patients between the ages of 19-69 years, from which 220 fulfilled the inclusion criteria. Each panoramic radiograph was analysed and the above three parameters recorded and measured. These values were collated into appropriate age and gender groups and subjected to statistical analysis.

**Results:**

The mean age of the participants was 44.1±14.41, with males being shown to have a statistically significant larger ramus height and bigonial width than females (*P*<0.0001 for both). Females, on the other hand, were shown to have a significantly larger gonial angle than males (*P*<0.0002). General trends revealed gonial angle to increase with age, whilst bigonial width and ramus height were shown to decrease with age.

**Conclusions:**

The assessment of mandibular morphology through radiographic measurements may be useful in estimating an individual’s age and gender when comparing to a known population standard.

** Key words:**Bigonial width, gonial angle, panoramic radiograph, ramus height.

## Introduction

Panoramic radiographs are commonly used in daily routine dental practices to assess mandibular and maxillary vital structures. They are a convenient radiologic approach to survey dental conditions by providing information about most aspects of dentistry using a single film. The high rate of prescription of panoramic radiographs means it is a useful tool to study the morphological changes that occur with age as well as any differences or correlations between genders. There have been numerous studies in the past decade that have proven the efficacy of Orthopantomograms for the determination of morphological dimensions of the mandible (1-6). Various studies (6-10) have utilized panoramic radiographs to measure three mandibular parameters, gonial angle, ramus height and bigonial width. The influence of individual age and gender on the degree of gonial angle is controversial (11,12). Some studies (11) have shown widening of gonial angle with advancement of age, others have reported conflicting results (1,12). There has also been differences in the gonial angle measurements in comparison to genders in some studies (1,11,12). Ohm and Silness (10) and Dutra *et al.* (13) found no significant difference in gonial angle between sexes.

In the past, lateral cephalograms were the radiograph of choice for measuring morphological changes to the mandible (12). However as they do not allow bilateral mandibular assessment due to the superimposition of the ramus, researchers have now looked to orthopantomograms for a more reliable method of obtaining data (1-6,12,14,15). Studies (3,14) published reveal that the gonial angle was the parameter with acceptable accuracy and precision in determining gender, which in turn suggests a forensic implication (14). From a medicolegal point of view, odontology is commonly used to identify human remains. Research into age determination from dental radiographs largely consists of the use of lateral cephalograms and orthopantomograms with the majority of papers investigating the gonial angle and few researching ramus height and bigonial width. Upadhyay *et al.* (9) suggest that gonial angle alone is not sufficient to determine age, as there are multiple factors that influence its development. For this reason, further research is required relating to other morphological characteristics of the mandible, in order to provide a more reliable indicator of age (9). Furthermore, the use of gonial angle, ramus height and bigonial width may be of great interest from an orthodontic point of view. Gonial angle is regularly used to determine the rotation of the mandible and to diagnose growth patterns (3). Gonial angle is a common parameter used to depict orthodontic extractions or surgical treatments (3). To date there has been limited research into age and gender differences in ramus height and bigonial width with only two studies noting any change in parameters in regards to gender (1,12). Joo JK *et al.* (16) found men have a higher value for ramus height in edentulous subjects. Internationally, studies have been conducted in an attempt to correlate age and gender with mandibular parameters but at present, there are no known studies conducted on the Australian population (10). The identification of these limitations in the current literature created the framework for the development of this research.

This study aimed to determine if there is a correlation between three mandibular parameters (gonial angle, ramus height and bigonial width) and age or gender in dentate subjects visiting a dental school in Far North Queensland. This data may enable future advances in forensic cadaver identification, as well as monitoring growth patterns of individuals in orthodontic assessments (3).

## Material and Methods

This study aimed to determine if there is a correlation between three mandibular parameters (gonial angle, ramus height and bigonial width) and age or gender in dentate subjects visiting a dental school in Far North Queensland. Panoramic radiographs of patients who attended the undergraduate or post graduate orthodontic clinics at James Cook University (JCU) between January 2011 and October 2014, were retrieved from records and were retrospectively evaluated. 2699 radiographs were selected randomly from the de-identified register held at JCU dental clinic and examined for eligibility against selection criteria. To ensure consistency, one investigator was responsible for the selection of radiographs based on the inclusion and exclusion criteria. Subjects with a natural permanent dentition (with the exclusion of third molars), and clear panoramic radiographs on which all structures are visible were included in the study. The participants were aged between 18 and 69 years inclusive, and were separated into five groups of ten year brackets. Completely edentulous and patients with oligodontia were excluded. It has been found that the reliability of panoramic radiographic technique for imaging of the mandible is highly dependent on patient head position (17,18). Hence for standardisation purposes, the radiographs included were only those taken by a radiographer on the same panoramic unit (Instramentarium® Model OP 100, Tuusula, Finland) considering standard exposure parameters (68Kv, 8mA, 18 sec, total infiltration 2.5mm AL, focal spot 0.3mm and magnification factor 1:1.1).

A low level negligible risk human research ethics approval was obtained from the James Cook University Ethics Research committee for the proposed research. The sample size was calculated using stata 13 software (StataCorp. 2013. Stata Statistical Soft-ware: Release 13. College Station, TX: Statcorp LP). The targeted sample size was calculated to be 220 participants with a minimum of 22 radiographs in each of the 5 groups and even gender distribution (220=22*5*2). A sample size of 220 (110 males and 110 females) provided adequate study power (1-beta=0.8, alpha (*p* value) = 0.05) to detect differences of at least 2.5 mm between females and males (two-tailed t test). This sample size also provided adequate study power to detect an average difference of at least 1.7 degrees between the right and left hand sides (paired t-test). Independent samples 2-tailed t-test were used to compare means of gonial angle, ramus height and bigonial width between different age groups.

**Figure 1:**
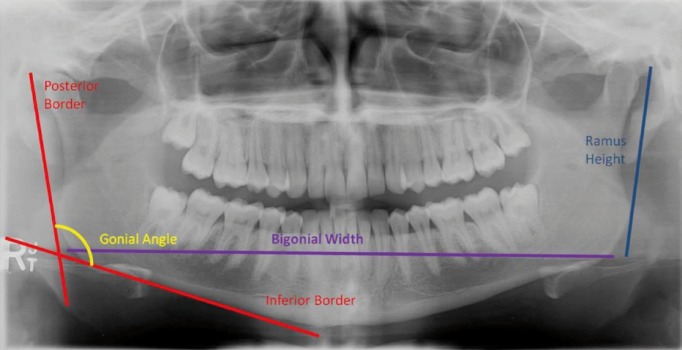
Measurements of the gonial angle, ramus width and bigonial width on panoramic radiographs.

Measurements were performed by five investigators with three measurements taken for each panoramic radiograph. Magnification compensation was performed using the magnification factor (10%) provided by the manufacturer. Sidexis software (Sirona; The Dental Company, 2012, Sidexis Digital Imaging Software. Sirona, Bensheim, Germany) was used to digitally trace lines on the panoramic radiographs. The gonion is the most inferior, posterior and lateral point on the external angle of the mandible. Gonial angle measurements were undertaken as described by Upadhyay *et al.* (9) measuring between 2 tangents from the gonion; the first running superiorly along the posterior border of the mandibular ramus and the other anteriorly along the inferior border of the body of the mandible (9). This was measured bilaterally on the left and right hand side of each radiograph in order to produce an average value. Ramus height was measured by a line drawn from the most superior point of the condylar head to the most inferior point of ramus tangent on both sides as described by Saini *et al.* (19). Bigonial width was measured horizontally between the left and right gonion as described by Lux *et al.* (20) (Fig. 1). The first 50 radiographs were measured by two investigators and intra-class correlation coefficient (ICC) for intra and inter-examiner reliability was found to be greater than 0.93 for all three parameters.

-Statistical Analysis 

The analyses were performed using SPSS for windows version 20 (IBM Corp. Released 2013. IBM SPSS Statistics for Windows, Version 20. Armonk, NY: IBM Corp.) Paired sample t-tests were carried out to compare values for the left and right hand sides (ramus height and gonial angle). Independent sample 2-tailed t-tests were used for male-female comparison of the ramus height, bigonial width and gonial angle, as well as for comparison between the different age brackets (frequency distribution plot showed normal distribution of results around the means). The level of significance was set at 5%.

## Results

2699 radiographs were evaluated, with 220 meeting the criteria for inclusion and analysis.  Table 1 illustrates the age and sex distribution of the subjects. The overall mean age was 44.1 ± 14.41 and although the average age for males was slightly higher, the difference was not statistically significant (*p*=0.9422).

**Table 1:**
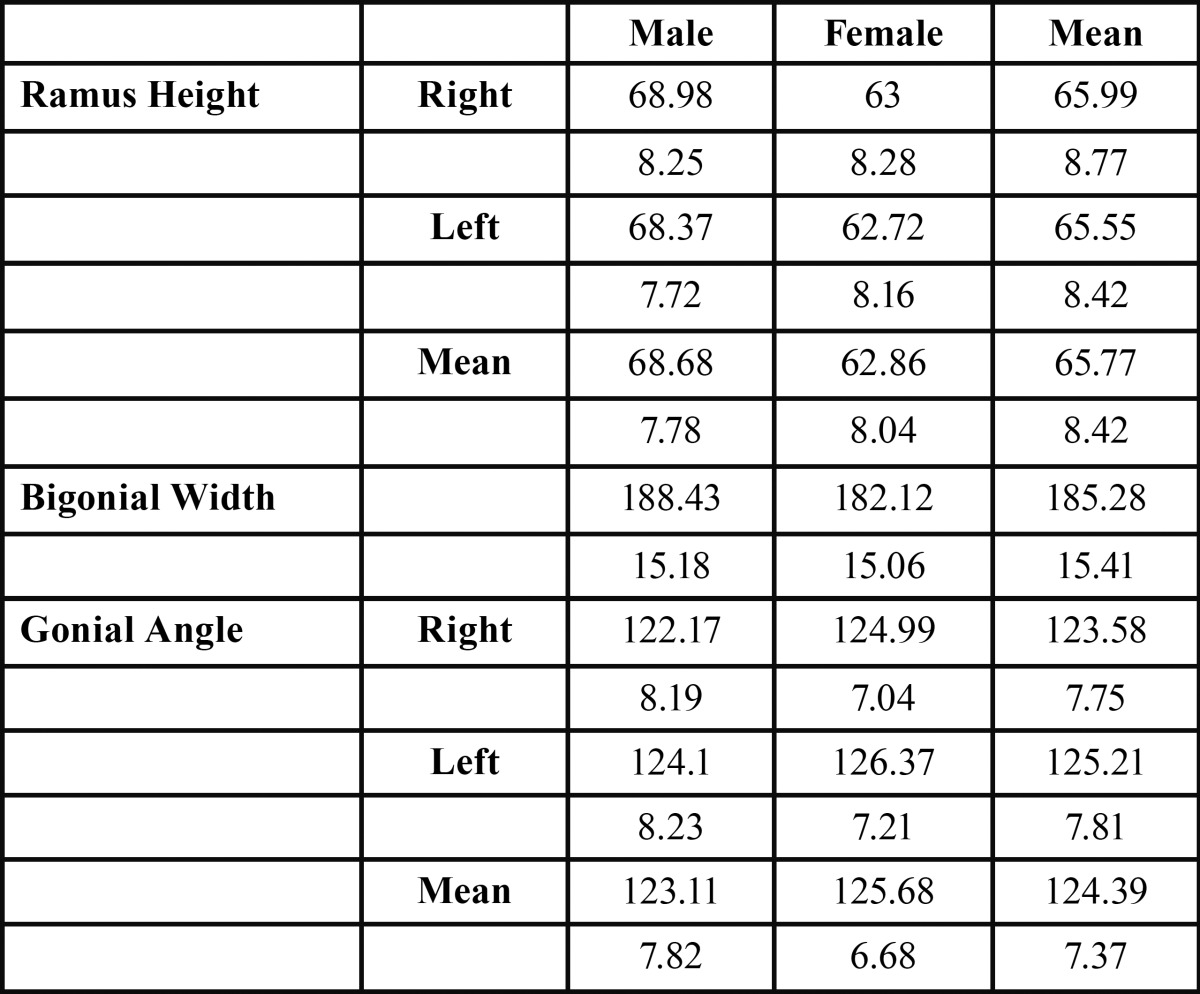
Gender differences in ramus height, bigonial width and gonial angle on right and left sides.

 Table 1 demonstrates gender differences in ramus height, bigonial width and gonial angle bilaterally. The mean of the ramus height was slightly higher on the right hand side and the gonial angle slightly higher on the left (68.98±8.25(R):M 68.37±7.72(L) and 122.17±8.19(R): 124.1±8.23(L) respectively); however, these differences were not statistically significant ( Table 2). Moreover, males showed higher values for ramus height and bigonial width than their female counterparts, and female values higher for gonial angle ( Table 1). Statistically significant gender differences were recorded for ramus height, bigonial width and gonial angle ( Table 3) (*P*<0.0001, *P*<0.0001 and *p*=0.0002 respectively; 2-tailed t test).

**Table 2:**
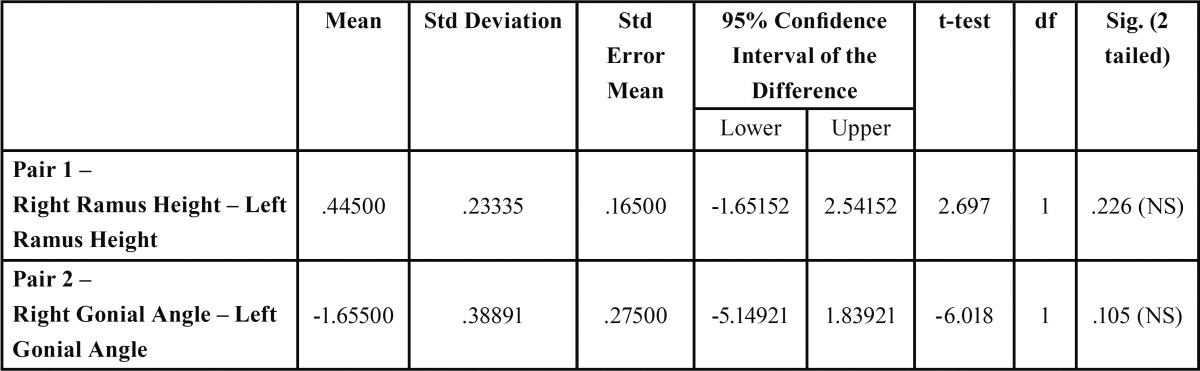
Paired T-test table for right-left comparison of ramus height and gonial angles.

**Table 3:**
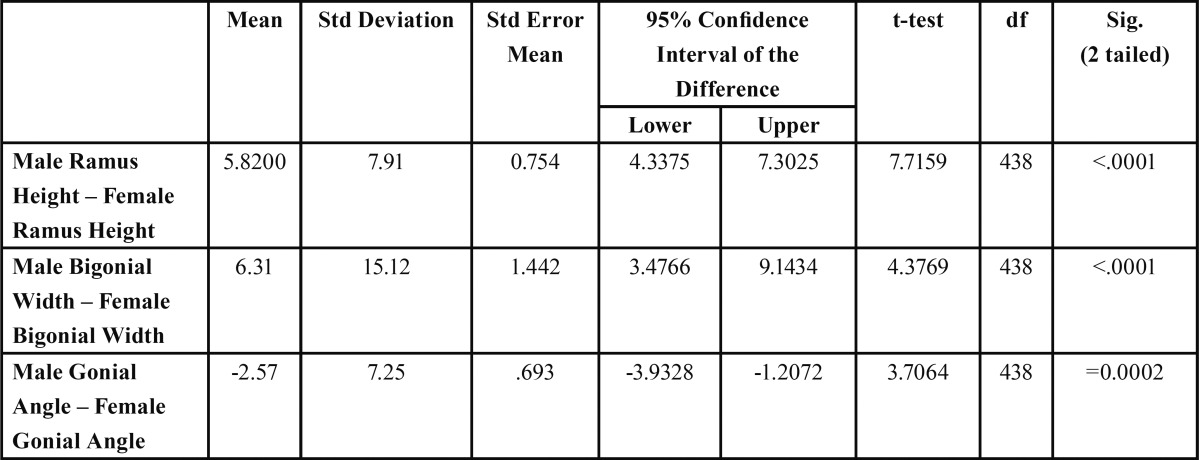
Independent samples T-test for male-female comparison of ramus height, bigonial width and gonial angle.

 Table 4 shows mean values of ramus height, bigonial width and gonial angle between 5 age brackets. Gonial angle increased with age and bigonial width decreased with age. Ramus height fluctuated between the ages of 18 and 40, showing a steady decline into the 5th and 6th decades. Independent samples t-test analysis, illustrated in  Table 5, showed a statistically significant difference in ramus height when comparing across all age brackets except for (30-39 compared to 50-59) and (30-39 compared to 60-69). Bigonial width also showed statistically significant differences across the board except for (19-29 compared to 30-39/40-49) and (50-59 compared to 60-69). Gonial angle, on the other hand, only showed statistically significant differences when comparing the 19-29 age bracket with the 40-49, 50-59 and 60-69 groups.

**Table 4:**
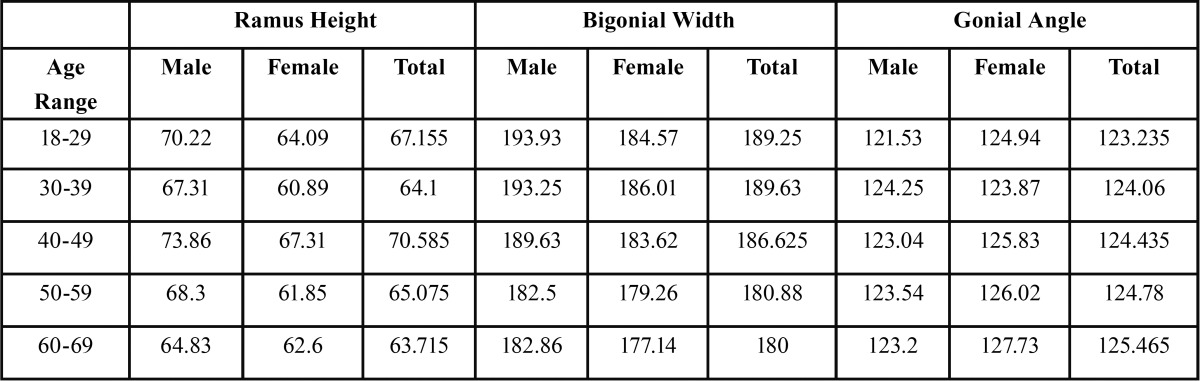
Mean values of ramus height, bigonial width and gonial angle related to age groups.

**Table 5:**
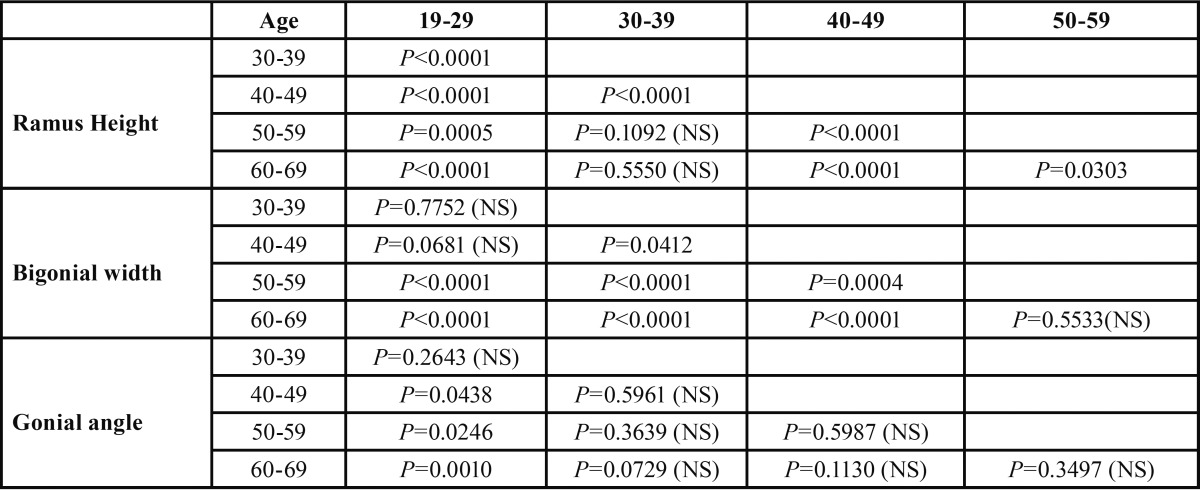
Independent 2-Tailed T-test for age group comparison of ramus height, bigonial width and gonial angle.

## Discussion

Investigations were carried out to determine if there was a correlation between three mandibular parameters and age and gender using measurements of gonial angle, bigonial width and ramus height in orthopantomogram radiographs in a Far North Queens-land population. Three parameters were examined, representing angular, vertical and horizontal dimensions. This allowed the morphology of the mandible to be observed and differences noted between sexes as well as observing the effects of aging and consequential mandibular remodeling. The investigations revealed a correlation in mandibular morphology in both gender and age. It was found that males have a larger ramus height and bigonial width than females, but a sharper gonial angle. A general trend in age showed a decrease in ramus height and an increase in gonial angle as age increased.

Several studies have found gonial angle to be the most accurate measurement obtainable from an orthopantomograph (1,2,4,6,7,21). This is most likely because it is an angular rather than linear measurement and as a result is not affected by the magnification factor (1,2,7). The study found no significant difference when comparing left and right gonial angles, regardless of gender. Females were found to have a significant higher value of gonial angle than their male counterpart; which was analogous to the results obtained by Ghosh *et al.* (22) and Joo JK *et al.* (16). However, our results were not in agreement with Dutra *et al.* (13), where no significant difference found between genders. There was a trend of gonial angle increase with age, but it was only significant when comparing the 19-29 age group with the older age groups (40-49, 50-59 and 60-69). This trend was also noted by Ghosh *et al.* (22) who concluded that the gonial angle increased with increase in age.

When comparing bilateral measurements of ramus height, no significant gender difference was found. Concurrent with the findings of Joo JK *et al.* (16), males were found to have a significantly higher ramus height than their female counterparts. There was fluctuation in ramus height with increasing age, with a steady decline in the 5th and 6th decades. This was significantly different between all age groups except when comparing 30-39 to 50-59 and 30-39 to 60-69.

Gender differences were statistically significant with males having a higher value of bigonial width than females. Our investigations, on the other hand revealed that bigonial width significantly decreased as age increased. However, it was not significant when comparing 19-29 to 30-39 or 40-49 and 50-59 to 60-69.

The study was hospital based and is limited to the Australian population and in particular, a small Far North Queensland population. Further research should be conducted across other areas and populations of Australia. Cone Beam computed tomography is a relative new technology introduced over the last decade (23). It gives accurate display of dimensions and could be an appropriate direction for future studies of age and gender differences of the mandibular parameters. Research should be performed including differing skeletal patterns and levels of edentulism to investigate changes in mandibular morphology.

The findings give a Far North Queensland reference of average measurements of the mandible and may be useful in orthodontic analysis or forensic identification. Gonial angle is a commonly used parameter to determine gender and hence can be used to identify human remains (9). Gonial angle is also regularly used to determine the rotation of the mandible and to aid in diagnosing growth patterns in order to depict orthodontic extractions or surgical treatments (2).

## Conclusions

Orthopantomograms have been proven to be a valuable tool for the determination of morphological dimensions of the mandible. Through the use of mandibular parameters such as gonial angle, ramus height and bigonial width, variations and correlations between age and gender can be examined. The implications of such correlations have numerous applications in the fields of forensic identification and orthodontic analysis. From the results obtained within the Far North Queensland population; several conclusions can be drawn. Males had a larger ramus height than females; however, on average females had the larger gonial angle. There was a steady decrease later in life in ramus height, with gonial angle generally increasing as the population aged. Bigonial width demonstrated a larger average value for males as opposed to females and a steady decrease with increasing age, although this decline was not consistently significant across all age groups. Future research should be conducted across a vast area of Australia to provide a more representative sample that accurately reflects the entire Australian population.
